# Minor surgery in general practice in Ireland- a report of workload and safety

**DOI:** 10.1186/s12875-020-01186-x

**Published:** 2020-06-23

**Authors:** Ailís ní Riain, Niall Maguire, Claire Collins

**Affiliations:** 1 Research Department, Irish College of General Practitioners, 4-5 Lincoln Place, Dublin 2, Ireland; 2 Primary Care Surgical Association, Navan, Ireland

**Keywords:** Minor surgical procedures, General practice, Dermatology, Skin cancer, Family medicine

## Abstract

**Background:**

The provision of minor surgical services is an established part of the task profile of general practitioners (GPs) in many countries in Europe and elsewhere. This study aimed to collect data on the clinical process and outcomes for specified minor surgical procedures undertaken in Irish general practice by GPs experienced in minor surgery in order to document the scope and safety of minor surgery being undertaken.

**Methods:**

Over a six-month period, 24 GPs in 20 practices recorded data on a pre-determined list of procedures undertaken in adults (aged 18 and older); procedures for ingrown toenails were also recorded for those aged 12–18 years. Clinical data were rendered fully anonymous by the participating GPs, entered onto the Excel database template and returned to the project team monthly.

**Results:**

On average, each practice undertook 212 procedures in a six-month period. The four most frequent procedures include two relatively non-invasive procedures (cryosurgical ablation of skin lesions and aspiration and/or injection of joints) and two more invasive procedures (full thickness excision of skin lesion and shave, punch or incisional biopsy). Overall, 83.8% of relevant specimens were submitted for histology. Combining benign and malignant cases, there was an overall 87% clinical and histological concordance; 85% of malignancies were suspected clinically. A complication was recorded in 0.9% after 1 month.

**Conclusions:**

Irish GPs with experience in minor surgery can provide a range of surgical services in the community safely.

## Background

The provision of minor surgical services is an established part of the task profile of general practitioners (GPs) in many countries in Europe and elsewhere [1]. Incentives and referral audit have been shown to influence the location of this sort of work in several countries [2] but much of the variation in service provision between countries reflects the medical culture and inherited boundaries to the scope of practice of the GP [1, 3]. The health, social and economic drivers to shift health care generally from the secondary to the primary setting [4] apply particularly to minor surgical procedures.

While GPs’ clinical diagnostic concordance with specialists is good for common inflammatory dermatoses such as acne, psoriasis and atopic dermatitis, it is poorer for the common skin malignancies (basal cell carcinoma (BCC), squamous cell carcinoma (SCC) and malignant melanoma) [5, 6]. Several studies from the United Kingdom (UK) have reported poorer quality of surgery for skin cancers in general practice compared to hospital settings [6–9], although one study showed that GPs compared favourably to skin specialists and outperformed hospital non-specialists in the excision of squamous cell carcinomas [10]. In studies from both the UK [11] and Ireland [12], the outcomes for primary excision of malignant melanoma were shown to be equivalent between GP and hospital providers. A community-based surgery audit from the UK, which examined 6138 procedures, concluded that GP minor surgery was safe and prompt and that GPs working within a managed framework performed better than less well-supported GPs [13].

Two thirds of Irish GPs have previously reported that they undertake minor surgical procedures [14] but the range, volume and outcomes of such procedures are unknown. The relatively high cost of undertaking minor surgical procedures in hospitals in Ireland has been established [15] and it is estimated that up to 60% of such procedures currently undertaken in hospitals could be performed in general practice [16]. The Health Service Executive (HSE), which is responsible for the delivery of public healthcare services in Ireland, funded a national pilot project to develop an accreditation process for minor surgery in general practice among GPs participating in the General Medical Services (GMS) scheme. Approximately 40% of Irish people are eligible for free GP care at the point of service (GMS patients); others may have private health insurance or are self-paying. The accreditation elaborated a series of standards against which the qualification, infrastructure and practice of GPs commissioned to undertake work devolved from hospitals would be measured and is described elsewhere [17].

This paper describes the surgical case mix and outcomes of the 24 GPs who were engaged in the accreditation process over 6 months in 2016. The aims of the research were to describe the range, frequency and inter practitioner variance in minor surgery and to measure the safety of their work.

## Methods

A multi-disciplinary project steering group drawn from general practice, nursing, pathology, plastic and general surgery agreed a designated list of procedures commonly undertaken by at least some GPs. This list reflected the procedures regularly undertaken in general practice in Ireland [14, 18, 19] (Additional file 1).

The project budget was restricted to twenty practice sites. Twenty-four GPs in 20 practices were recruited by open competition following a national advertisement. Seventy-two GPs in 64 practices applied. They were eligible for consideration if they met personal criteria (post graduate membership examination, GMS contract holder); practice criteria (suitable premises and equipment, a surgical assistant, a procedures log, a health and safety statement); and workload criteria (performed three of four core procedures of shave and punch biopsy, excisions of cysts or lipomas, excisional biopsy of skin and excision of ingrown toenails, had undertaken at least 50 procedures in the past 6 months).

Thirty applications (34 GPs in 30 practices; 27 individual and 3 group applications) met the eligibility criteria. Among these, 24 GPs in 20 practices were selected to ensure geographic spread and a mix of urban and rural sites. None were surgically trained beyond Senior House Officer grade, and it was felt that they were reflective of the experienced GP in Ireland. Each participating practice received a payment of €15,000 in stages over 12 months to enhance the extant modest payments for GMS patients (€25.00 per case) available to incentivize provision of these services and to cover engagement in the accreditation and research elements of the project.

Over a six-month period, GPs recorded data on all the designated procedures undertaken in adults (aged 18 and older) as well as procedures for ingrown toenails for those aged 12–18 years.

The steering group developed an Excel spreadsheet (Additional file 2) to record demographic and clinical data on patients having any of the agreed minor surgical procedures at participating sites. Demographic data included age and gender of the patient, their insurance status and whether they were patients of the operating GP or had been referred from another GP. Clinical data included the clinical diagnosis, location of the lesion, procedure undertaken, post-procedure complications, whether a specimen was sent for histological examination, histology result (where applicable) and the extent of cancer resection margins. Anonymous data were entered as free text in the spreadsheet and returned to the project team monthly. Follow-up data, such as the histological result and post-procedure complications, were submitted in the subsequent month.

Safety was assessed by reference to the frequency and type of surgical complications, the comprehensiveness of submission of all appropriate specimens for histological examination, the accuracy of the GP’s clinical diagnosis compared with the histology, the completeness of surgical excision of skin cancers and the avoidance of unnecessary excisions of benign lesions. Any full thickness excision of skin or subcutis was expected to be sent for histological assessment. Five of the studied procedures should routinely result in the submission of a specimen for histological examination. We regarded excisions with peripheral margins of 1 mm or less as inadequate following the British Royal College of Pathologists (RCPath) guidance for basal (BCC) [20] and squamous cell cancer (SCC) [21]. These parameters are established indicators of quality described in earlier evaluations of community surgery [6, 7, 13, 14, 22–25]. Complications were according to those reported to the GP within 1 month of the procedure; GPs were prompted to record the existence of complications and the type of complication.

## Results

Data were returned on 4263 procedures completed by the 24 GPs in the 20 research practices over 6 months. Twenty cases were excluded where no procedure type was given (*n* = 4), the procedure type was unclear (*n* = 5) or where the procedure was not part of the project schedule (*n* = 11). Of the 4243 procedures analysed, those most often carried out were cryosurgery of skin lesion, full thickness excision of skin lesion and joint aspiration/injection, accounting together for 65.5% of the total (Table 1). The frequency of procedures ranged between 80 and 588 among the participating practices with a mean of 212 procedures per practice.

**Table 1:** Procedures carried out by the participating GPs (*n* = 4243) over 6 months

Practice	Cryosurgical Ablation	Excisional biopsy	Joint Injection	Shave,Punch & Incisional biopsy	Therapeutic Phlebotomy	IGTN surgery	Curettage & Diathermy	_Incision_ & drainage	Excision epidermoid cyst	Suture laceration	Aspiration Ganglion	Enucleation lipoma	Excision Meibomian cyst	Total per practice
**1**	46	43	30	12	13	13	1	6	2	4	1	0	1	172
**2**	54	54	60	38	56	21	7	8	5	7	2	0	0	312
**3**	137	38	111	38	53	24	0	0	15	1	0	5	1	423
**4**	12	70	1	10	0	1	18	1	3	1		0	0	117
**5**	50	55	23	6	13	5	0	1	1	5	0	0	0	159
**6**	82	22	50	6	5	3	0	6	3	9	3			189
**7**	93	10	52	15	10	7	4	3	0	3	0	0	0	197
**8**	297	28	5	153	4	30	39	25	1	3	3	0	0	588
**9**	10	60	15	1	11	11	0	12	0	1	0	1	0	122
**10**	54	39	66	15	6	15	5	8	0	4	1	0	0	213
**11**	31	14	35	3	0	11	7	26	5	13	1	2	0	148
**12**	28	9	33	3	0	6	28	0	11	5	3	4	3	133
**13**	33	0	41	9	31	2	2	6	1	8	3	1	3	143
**14**	18	83	6	62	2	10	14	0	22	0	0	4	1	222
**15**	42	17	49	30	1	3	2	11	9	6	0	0	1	171
**16**	53	22	27	0	17	6	0	2	3	3	5	0	1	139
**17**	2	38	22	10	16	17	0	0	0	3	0	0	0	108
**18**	102	42	7	3	0	34	0	6	17	3	2	0	2	218
**19**	5	38	11	2	9	4	3	6	0	1	1	0	0	80
**20**	220	33	27	24	0	9	34	8	30	1	1	2	0	389
Total Procedures(n)	1369	718	671	440	247	232	164	135	128	81	26	19	13	**4243**
Total Procedures(%)	32.3	16.9	15.8	10.4	5.8	5.5	3.9	3.2	3	1.9	0.6	0.4	0.3	100

Overall, patient age ranged from 12 to 99 years (mean 55.6 years). Excluding surgery for in-grown toenails (IGTN), age ranged from 18 to 99 years (mean 56.6 years). For IGTN surgery, age ranged from 12 to 87 years (mean 39 years). Overall, 46.5% of patients were female and 53.3% were male. Nine cases did not have gender recorded. GMS patients made up 55.4% of those treated. Half of the procedures (*n* = 2149; 50.7%) were carried out on patients of the operating GP. An additional 22% were on patients of another GP in the same practice and 21.8% were on patients of a GP from another practice. Males were more likely to have had enucleation of lipoma, therapeutic phlebotomy for haemochromatosis, suture of lacerations and excision of epidermoid cysts. Women were more likely to have a ganglion aspirated. The remaining procedures showed no gender difference.

### Indicators of safety

#### Diagnostic accuracy

A histology report was available for 1131 cases. An initial clinical impression was recorded for 1016 (90%) of these patients. In 87% of cases, the preoperative diagnosis was confirmed on histology.

There were 284 (6.7%; 284/4242) histological reports of malignant or pre-malignant disease equating to 25.1% (284/1131) of cases where a histological report was stated to have been received. The clinical diagnosis was of a suspected malignancy in 228 cases (80.2%), of a benign disease in 40 cases (14.1%) and no clinical impression was recorded in the remaining 16 cases (5.7%). The sensitivity of the GP’s clinical impression for diagnosis of malignancy was therefore 85% and the specificity was 88%.(Table 2).

**Table 2:** Concordance between clinical impression and histological diagnosis (*n* = 1016*)

	Histological diagnosis N (% of total)		
Clinical diagnosis			Total
	Malignant/Premalignant	Benign	
Malignant/Premalignant	228 (22.4%)	92 (9.1%)	**320**
Benign	40 (3.9%)	656 (64.6%)	**696**
**Total**	**268**	**748**	**1016**

Sensitivity = 228/(228 + 40) = 0.85 Specificity = 656/(656 + 92) = 0.88

*No clinical impression was recorded for 115 cases and hence no comparison possible

The majority of the malignancies (269/284, 94.7%) were non-melanoma skin cancers (NMSC) or NMSC in-situ (Table 3). The face was the most common site of excised malignant lesions (108/284; 38%) with a further 15% (43/284) located on the remainder of the head and neck. There were 142 excisions of skin malignancies; six were treated by cryosurgery and 136 were biopsied (Table 3). Subsequent management of the cases biopsied was not part of this study. Twelve patients were treated for two malignancies and five for three during the six-month data collection period.

**Table 3:** Malignant diagnoses on histological reports (*n* = 284)

		Initial management		
Histological diagnosis	Frequency (%)	Excision	Biopsy only	Cryosurgery
Basal cell carcinoma	116 (40.9%)	69	43	4
Squamous cell carcinoma	56 (19.7%)	30	26	0
NMSC-type not specified	48 (16.9%)	19	28	1
In-situ NMSC	49 (17.3%)	16	32	1
Melanoma*	8 (2.8%)	5	3	0
Melanoma in-situ*	3 (1%)	3	0	0
Cutaneous lymphoma	4 (1.4%)	0	4	0
**Total**	**284 (100%)**	**142**	**136**	**6**

*NMSC*non-melanoma skin cancer

#### Submission of tissue for histology

Five of the procedures undertaken should routinely have specimens sent for histological examination, totaling 1469 procedures (34.6% of all procedures) (Table 4). Overall, 83.8% of these procedures had specimens submitted for histology, with 77% having the histology result documented at the time of submission of the research data. Doctors were more likely to send specimens for histological examination where they had undertaken full thickness excisions of skin lesions (96.7%) and shave, punch and incisional biopsies (91.9%). They were least likely to do so in the cases of curettage and cautery (29.3%).

**Table 4:** Frequency of specimens submitted for histology in appropriate procedures (*n* = 1469)

Procedure	Not submitted (% of total for the procedure)	Submitted (% of total for the procedure)	Histology report recorded (% of total for the procedure)	Total number
Full thickness excision of skin lesion	24 (3.7%)	714 (96.7%)	668 (90.5%)	738
Shave, punch and incisional biopsy of skin lesion	34 (8.1%)	386 (91.9%)	358 (85.2%)	420
Curettage and cautery	116* (70.7%)	48 (29.3%)	41 (25%)	164
Excision of epidermoid cyst	61 (47.7%)	67 (52.3%)	51 (39.8%)	128
Enucleation of lipoma	3 (15.8%)	16 (84.2%)	13 (68.4%)	19
**Total**	**238 (16.2%)**	**1231 (83.8%)**	**1131 (77%)**	**1469**

*Includes 15 who responded “not applicable”

#### Completeness of excision of skin malignancy

For the 142 malignancies which were excised, a measurement of the least peripheral margin of excision was available in 93 cases (65%). A qualitative report of the lateral margin was available in a further 10 cases, of which two were reported to be tumour involved. In 24 cases, the data returned had neither deep nor lateral margin as a measurement or as a qualitative grade. The number of peripheral margins reported as less than 1 mm was 3/93 (3.2%) while 13/93 (14%) had margins of 1 mm.

#### Inappropriate excision

Where a full thickness skin excision was undertaken as treatment of a benign lesion amenable to less invasive treatment, the procedure was considered to be a possibly inappropriate excision. There were 71 such cases among the 887 procedures for which clinicopathological correlation was possible (8%). The clinical diagnosis was correct in 41 cases (57.7%). The histology in these 71 cases were seborrheic keratosis, wart and fibroepithelial polyp.

#### Surgical complications

A complication was recorded in 40 of the 4243 procedures (0.9%) within 1 month of the procedure. No complications were reported for five of the procedures studied (Table 5).

**Table 5:** Complications reported for minor surgical procedures

	Frequency	Number of complications (% for procedure)	Complication
Cryosurgical ablation of skin lesions	1369	9 (0.7%)	Infection (5)
			Other (4)
Full thickness excision of skin lesion	738	20 (2.7%)	Infection (5)
			Dehiscence (3)
			Bleeding (2)
			Other (10)
Aspiration and/or injection of joint	670	2 (0.3%)	Other (2)
Shave, punch & incisional biopsy of skin lesion	420	3 (0.7%)	Infection (3)
Therapeutic phlebotomy	247	0	
Surgery to ingrown toenails	232	3 (1.3%)	Infection (3)
Curettage & cautery	164	0	
Incision & drainage of abscess or haematoma	135	1 (0.7%)	Bleeding (1)
Excision of epidermoid cyst	128	0	
Suture of laceration	81	1 (1.2%)	Infection (1)
Aspiration of ganglion	26	0	
Enucleation of lipoma	19	1 (5.3%)	Bleeding (1)
Incision & drainage of Meibomian cyst	13	0	
**Total**	**4242**	**40 (0.9%)**	

## Discussion

### Summary of results from this paper

The procedures studied in this project are those commonly undertaken by GPs anywhere [2, 13, 14, 18, 19, 26–32]. We selected participants who had sufficient experience to demonstrate the capacity of GPs in Ireland to perform minor surgery and to have a sufficient established scope of practice to test an accreditation procedure for community surgery [17]. This was a purposefully heterogenous group of GPs with a seven fold difference between the numbers of cases managed by the busiest and least busy practices in terms of minor surgery activity. Inter-practice referrals were common.

We report on key quality indicators of safety: submission of tissue for histological examination, accuracy of clinical diagnosis, completeness of excision of malignancy, avoidance of unnecessary excisions and surgical complications. On each of these measures, the participants had results in line with available comparable data [7, 13].

### Interpretation of results in light of literature

There has been a debate as to the scope of the GP’s practice and it is especially evident in the area of technical procedures [33]. Policies tending to devolve hospital surgical work to primary care, such as that underpinning the present project, have been seen in other jurisdictions [34].

There has been little argument that minor surgical services provided in the community are both convenient and cost effective [24, 27] and encouraging community provision does not invariably divert work from hospitals as much as it generates new activity, albeit to address an unmet need [24, 34].

The role of the GP in managing skin cancers has been resisted by specialist providers out of concern about safety [22, 23, 25, 35] or on health economic grounds [36–38].

The data we present represents the largest report of surgical work by GPs in Ireland.

Previous Irish surveys have established the range of procedures that are commonly or sometimes provided in general practice [18, 19]. These were the procedures commissioned in the current project and reflect the scope of primary care surgery internationally [2, 24, 28, 29, 32].

The variation between respondents in the numbers of cases reflects practice size and location.

O’Kelly reported that 65% of GPs in Ireland in 2015 stated that they provide some minor surgical services [14]. Earlier Irish surveys by Clarke [18] and White [19] indicate that a third of GPs frequently undertake the more common procedures we have studied. Barriers to wider provision in the community reported internationally, and likely at play in Ireland, include limited training, lack of time, equipment and insufficient compensation [39–41]. In establishing the present project, we were limited by budget rather than any difficulty attracting volunteers.

Overcoming the barriers that do exist to the provision of procedures in primary care would seem to require incentivization of the GP. In the course of this project, participants were compensated by way of enhanced income for their surgical clinics and by the achievement of accreditation. An economic assessment of whether this is the best way to supply patient demand for minor surgery was beyond our remit. On the other hand, the project arose from a concern as to the inordinate unit cost of provision in Irish hospitals [15, 16].

Economics aside, any commissioner of a devolved surgical service will have a concern as to safety. Quality assurance in minor surgery has many dimensions, including the doctor’s qualification, maintenance of professional competence, the quality of their premises, equipment and policies, as well as their clinical and technical ability.

Qualifications, infrastructure and governance are domains of high quality community surgical practice. These domains underpinned the accreditation standards, which have been reported separately [17].

In this study, we had the opportunity to measure the safety of the surgical service from the perspective of clinical and technical proficiency in relation to the cases undertaken as part of that accreditation procedure.

The key indicators of quality which we studied are established measures in minor surgical practice [6, 7, 13, 14, 22–25].

The diagnostic accuracy of the GP is important because better clinical diagnosis avoids unnecessary excisions as well as missed diagnoses of skin malignancy. This is measured by the clinicopathological correlation. Two aspects are of interest. The overall proportion of cases where the GP’s clinical diagnosis is confirmed on histology and the occurrence of an unrecognized malignancy are simple measures which can be audited.

It is well established and entirely expected that skin specialists (dermatologists and plastic surgeons) perform better at pre-operative diagnosis than either GPs or general surgeons [22, 25, 35, 42]. Early British reports from single pathology laboratories following the contractual encouragement of GP skin surgery in 1991 indicated alarmingly low rates of correct clinical diagnosis in GP specimens [25, 42]. In a British randomised trial of GP minor surgery, 45% of clinical diagnoses were confirmed at histology [6]. Overall clinicopathological concordance for all lesions among our cases was reassuring by comparison.

In relation to the clinical suspicion of malignancy in particular, our results compare well with recent audit data from the United Kingdom [13], the small number of malignant lesions included in the only extant randomized trial of GP minor surgery, and Corwin’s results from New Zealand [22]. The anxiety that the GP surgeon is missing skin cancer has been a long-standing concern of specialist surgeons and dermatologists [6, 25, 42]. However, this concern could apply equally to GPs and non-specialist skin surgeons who refer rather than operate.

Some unanticipated histological results are inevitable whatever the surgical setting. For this reason, adherence with the requirement that all excised material be studied by the pathologist is a further key indicator of safe practice. The majority of lesions eligible for histology were referred appropriately by the GPs in our study and accord with other reports [7, 13]. Though the ideal is that all excised tissue be submitted, in practice, GPs frequently do not submit small lesions such as skin tags and warts or grossly recognizable lesions such as sebaceous cysts and seborrheic keratoses [6]. These are commonly treated by curettage or shave excision. Our results appear to reflect that practice.

Where skin cancers are excised by the GP, the adequacy of resection margins is an important indicator of quality and a proxy for cure. Comparisons are difficult as studies often report a categorical assessment rather than one based on measurement or do not state how margins were assessed at all. We have only reported data relating to peripheral margins as deep margins are not reliably reported in our sample or generally in Ireland [43]. In adopting the RCPath guidance [20, 21], which regards a margin of 1 mm or less as being involved, we provide some indication of the completeness of excision. Internationally, much of the evidence indicates a greater likelihood of adequate excision of malignancy by specialist surgeons [7, 23, 44]. However, GPs may perform as well as non-skin specialist surgeons in excising BCC [43] and in one Scottish study, GPs of varying levels of experience had rates of complete excision as good as those of skin specialists [10].

However, prognosis is based on the clinical margin rather than the histological outcome and we did not study this [45]. Furthermore, a close margin in a BCC at a low risk site is a different prospect than the same margin for an aggressive subtype of SCC at a high risk site. Because of these issues, both our study and results reported elsewhere from similar surveys are inevitably of limited value in determining safety. Ideally, a randomised comparison of tumour recurrence rate over at least 5 years would be required to compare the GP and skin specialist outcomes.

An additional indicator of quality which relates to clinical diagnostic ability and surgical judgement is the rate at which benign lesions are excised as full-thickness skin resections. It is generally inappropriate to undertake surgical excisions of warts, skin tags, seborrheic keratoses other than by curettage, electrocautery or cryoablation. Our results for possibly inappropriate excisions compare favourably with those of a recent review of pathology requests at a regional laboratory in one Irish region [36].

A final and important measure of safety is the rate of surgical complication. Due to the limited length of follow-up, which extended to 1 month after the last recruitment, late complications such as poor scar and tumour recurrence are not assessed in our study. Our results are similar to those in Botting’s audit of over 6000 cases in Britain [13].

### Limitations

Besides the limitations relating to quality indicators already referred to, several general limitations pertain to these descriptive data.

The study was not primarily designed as a test of the quality and safety of Irish community surgery but as a description of work undertaken in the course of the design and piloting of a community surgery accreditation process, and this limits the strength of conclusions that can be drawn from our findings.

The requirement of the GPs to undertake double data entry is less than ideal when seeking to collect comprehensive details of surgical work, and structured clinical records linked to automated data extraction may be a better solution. This limitation may have resulted in missing or miscoded data.

The participating GPs were chosen on the basis of their surgical experience from a self-selected group of volunteers. Their performance cannot be taken to represent all Irish GPs. Nevertheless, minor surgery is provided by volunteers within general practice and our cohort may be deemed an appropriate representation of what the more experienced among them can achieve. However, the number of cases may be higher among this group than among the average general practitioner.

## Conclusions

The number and types of cases reported in this study, along with satisfactory performance on a range of accepted indicators of quality, indicate that Irish GPs with an established interest in skin surgery could undertake some of the surgical work which otherwise takes place in hospitals.

A health economic assessment of a shift to primary care surgery is lacking. The question as to the adequacy of diagnosis and treatment for skin cancers cannot be definitively addressed by this sort of descriptive study. A randomized trial with a sufficient duration of follow-up to identify recurrence and late complications is required.

## Supplementary information

**Additional file 1.** List of Procedures

**Additional file 2.**

## Data Availability

The data is available on reasonable request to the corresponding author.

## Abbreviations

BCC: Basal cell carcinoma
GMS: General Medical Service
GP: General practitioner
HSE: Health Service Executive
IGTN: In-grown toenail
NMSC: Non-melanoma skin cancer
RCPath: Royal College of Pathologists
SCC: Squamous cell carcinoma
UK: United Kingdom
