# Supplementary material for: Minor surgery in general practice in Ireland- a report of workload and safety
Source: BMC Fam Pract. 2020 Jun 23;21:115. doi: 10.1186/s12875-020-01186-x (PMC7310463; doi:10.1186/s12875-020-01186-x)
Supplement: Supplementary file 1 — Additional file 1. List of Procedures [file 12875_2020_1186_MOESM1_ESM.docx]

**Appendix 1: List of Procedures**

Cryosurgical Ablation

Excisional biopsy

Joint Injection

Shave, Punch & Incisional biopsy

Therapeutic Phlebotomy

IGTN surgery

Curettage & Diathermy

Incision & drainage

Excision epidermoid cyst

Suture laceration

Aspiration Ganglion

Enucleation lipoma

Excision Meibomian cyst
